# Preventive Medicine for Person, Place, and Planet: Revisiting the Concept of High-Level Wellness in the Planetary Health Paradigm

**DOI:** 10.3390/ijerph16020238

**Published:** 2019-01-16

**Authors:** Susan L. Prescott, Alan C. Logan, David L. Katz

**Affiliations:** 1 Department of Paediatrics, School of Medicine, University of Western Australia, Perth, WA 6009, Australia; 2 The ORIGINS Project, Telethon Kids Institute, Perth Children’s Hospital, Nedlands, WA 6009, Australia; 3 inVIVO Planetary Health, Research Group of the Worldwide Universities Network (WUN), West New York, NJ 10704, USA; aclnd@cfs-fm.org; 4 Prevention Research Center, Yale University School of Public Health, Griffin Hospital, Derby, CT 06418, USA; davkatz7@gmail.com

**Keywords:** planetary health, preventive medicine, clinical ecology, social justice, health disparities, ecosystems, non-communicable diseases, dysbiosis, natural environments, vitality and sustainability

## Abstract

Experts in preventive medicine and public health have long-since recognized that health is more than the absence of disease, and that each person in the ‘waiting room’ and beyond manifests the social/political/economic ecosystems that are part of their total lived experience. The term planetary health—denoting the interconnections between the health of person and place at all scales—emerged from the environmental and preventive health movements of the 1970–1980s. Roused by the 2015 Lancet Commission on Planetary Health report, the term has more recently penetrated mainstream academic and medical discourse. Here, we discuss the relevance of planetary health in the era of personalized medicine, gross environmental concerns, and a crisis of non-communicable diseases. We frame our discourse around high-level wellness—a concept of vitality defined by Halbert L. Dunn (1896–1975); high-level wellness was defined as an integrated method of functioning which is oriented toward maximizing the potential of individuals within the total lived environment. Dunn maintained that high-level wellness is also applicable to organizations, communities, nations, and humankind as a whole—stating further that global high-level wellness is a product of the vitality and sustainability of the Earth’s natural systems. He called for a universal philosophy of living. Researchers and healthcare providers who focus on lifestyle and environmental aspects of health—and understand barriers such as authoritarianism and social dominance orientation—are fundamental to maintaining trans-generational vitality at scales of person, place, and planet.

## 1. Introduction

*HEALTH: i. The state of an animal or living body, in which the parts are sound, well organized and disposed, and in which they all perform freely their natural functions; in this state the animal feels no pain; this word is also applied to plants. ii. Sound state of the mind; natural vigor of faculties. iii. Sound state of the mind in a moral sense; goodness.*

Health as defined in Scientific Dictionary, 1863 [1]

Viewed through the prism of life (Greek; bios) and ways of living (Greek; biosis), health is an expansive term which has long-since defied concrete definition. In 1946, the World Health Organization’s constitutional statement [2] maintained that health is ‘complete physical, mental and social wellbeing’. However, the front-facing view of health—and the dominant efforts of professionals described as ‘healthcare’ workers—remains focused on the treatment of pathology and disease prevention. 

Gains to civilization by effective disease eradication/prevention through biomedicines, vaccines, surgical techniques, and public health measures have been immense. However, perhaps because of these successes, the word health (its attainment and maintenance) continues to be conflated with the absence of disease. This seems especially so when beheld against the crisis-level challenge of non-communicable diseases (NCDs) which have been less responsive to the armamentarium of westernized biomedicine. The focus on wellbeing, or wellness, in the definition of health—and prevention of the loss of vitality—has been diluted and overlooked.

**Figure 1:**
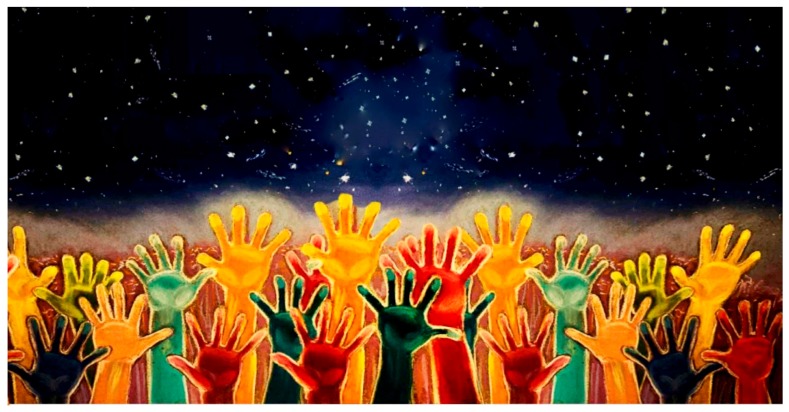
High-level wellness is applicable to organizations, communities, nations, and humankind as a whole. In an era of gross environmental concerns and a crisis of non-communicable diseases, personalized medicine must be increasingly viewed in the context of planetary health [image by author, S.L.P.].

In a landmark paper in the *American Journal of Public Health* (1959), biostatistician and public health physician Halbert L. Dunn urged professionals to move beyond the static notion of ‘health’ and conceptualized the idea of ‘high-level wellness’ (Figure 1). He proposed that high-level wellness—as a goal and way of life—was applicable at scales of person, communities, and civilization at-large; he defined high-level wellness for the individual “*as an integrated method of functioning which is oriented toward maximizing the potential of which the individual is capable, within the environment (in which they) are functioning*” [3]. In simplest form, the goal of high-level wellness was life with energy, vitality and zest, and it could only be concretized as a ‘way of life’ [4]. 

Remarkably—even without our current, sophisticated understanding of biodiversity losses, environmental degradation, climate change, and resource depletion—Dunn underscored that high-level wellness is predicated upon the health of the Earth’s natural systems [5]. In other words, discussions of high-level wellness—whether for person or civilization—must always consider the environment, and this must include broad aspects of the natural environment on which humans depend. Dunn was underscoring the principles of what is now termed ‘planetary health’. 

**Figure 2:**
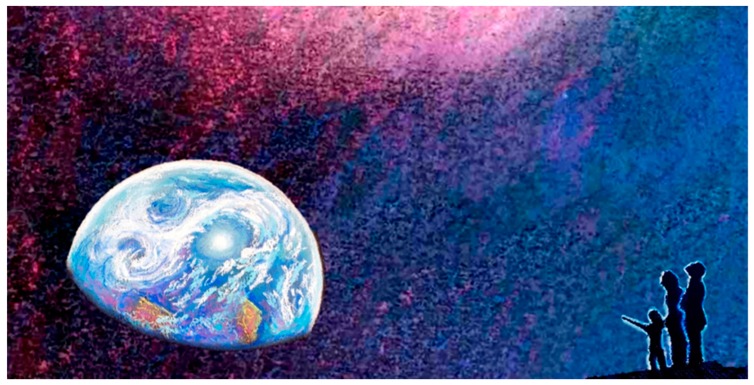
Striving for global high-level wellness requires restoration and preservation of the vitality and sustainability of the Earth’s natural systems—a universal philosophy of living, as advocated by Halbert Dunn [8]. [image by author, S.L.P.].

The term planetary health, popularized in the 1980–1990s, underscores that human health is intricately connected to the vitality of natural systems within the Earth’s biosphere. Coincident with the rise of environmentalism, preventive medicine and the self-care movements of the 1970s, the artificially drawn lines between personal, public, and planetary health began to diminish [6,7] Dunn’s concept of high-level wellness was referenced in articles which discussed “*a different philosophical framework through which individual, community, environmental and planetary health can be better understood in a broad and integrated fashion*” [8] (see Figure 2). 

As the global health burdens have shifted from infectious to NCDs, greater emphasis has been placed on the health-mediating role of social determinants, lifestyle, and the total lived environment. The health implications of anthropogenic threats to life within the biosphere cannot be uncoupled from discussions of the individual, community, and global health. Recent endeavors such as the Lancet Commission on Planetary Health [9] and The Canmore Declaration [10] have re-emphasized that public health, biopsychosocial medicine, and planetary health are one-and-the-same. 

## 2. Roadmap to the Current Review

Here in our narrative review, we will revisit Dunn’s high-level wellness and explore its place in the emerging planetary health paradigm. First, we discuss some of the origins of the high-level wellness concept and describe how it manifests in contemporary clinical care. Next, we examine the concept of planetary health, its historical origins, and the global movement which now considers the health of civilization and the Earth’s natural systems as inseparable. With this background in place, we argue that the concept of high-level wellness provides an essential framework for health promotion and clinical care in the modern landscape; it allows scientists of diverse fields—no matter how reductionist the scope of their inquiry—to see the large-scale relevancy of their work; it provides healthcare providers a broader vision of human potential with individuals as living embodiments of accumulated experiences shaped by natural and anthropogenic (i.e. social, political, commercial etc.) ecosystems—rather than a vision limited to a neutral disease-free set point. 

Dunn’s high-level wellness and planetary health (which we argue are synonymous) requires discourse concerning values, our connectedness to one another, our sense of purpose/meaning, and our emotional connections to the natural world. High-level wellness also demands discussion of authoritarianism, social dominance orientation, narcissism, and other barriers to vitality of individuals, communities and the planet. Finally, we emphasize that experts in environmental health promotion and lifestyle medicine are ideally positioned to educate and advocate on behalf of patients and communities (current and future generations), helping to promote vitality and safeguard the health of person, place, and planet. 

## 3. High-Level Wellness

*“Wellness is conceptualized as dynamic—a condition of change in which the individual moves forward, climbing toward a higher potential of functioning. High-level wellness for the individual is defined as an integrated method of functioning which is oriented toward maximizing the potential of which the individual is capable, within the environment where (they) are functioning. This definition does not imply that there is an optimum level of wellness, but rather that wellness is a direction in progress toward an ever-higher potential of functioning…high-level wellness, therefore, involves (1) direction in progress forward and upward towards a higher potential of functioning, (2) an open-ended and ever-expanding tomorrow with its challenge to live at a fuller potential, and (3) the integration of the whole being of the total individual—(their) body, mind, and spirit in the functioning process…high-level wellness is also applicable to organization, to the nation, and to (humankind) as a whole”.*

Halbert L. Dunn, MD, PhD. Canadian Journal of Public Health, 1959 [11]

In two notable papers—both published in 1959 [3,11]—biostatistician and public health physician Halbert L. Dunn conceptualized the idea of ‘high-level wellness’ (Box 1) for humankind and civilization at-large, maintaining that “*wellness is not just a single amorphous condition…but is rather a fascinating and ever-changing panorama of life itself, inviting exploration of its every dimension*” [3]. In this context, he included population pressures, rising rates of mental and functional illnesses, and the rapid speed of technological growth (especially in communications). Moreover, he stated: “*it is probably a fallacy for us to assume, as so many of us have done, that an expansion in scientific knowledge can indefinitely counterbalance the rapidly dwindling natural resources of the globe*” [3]. In other words, Dunn was acutely aware, even in 1959, that the ability to obtain high-level wellness—at individual and civilization-wide scales—was predicated on the health of the planet.

Box 1 **High-Level Wellness and Planetary Health.**

“High-level wellness is applicable not only to the individual but also to all types of social organizations—to the family, to the community, to groups of individuals, such as business, political or religious institutions, to the nation and to (humankind) as a whole. For each of these aggregates, it implies a forward direction in progress, an open-ended expanding future, interaction of the social aggregate and an integrated method of functioning which recognizes the interdependence of (humans) with other life forms”.

Halbert L. Dunn, MD, PhD. 1966 [12]

Dunn’s context for high-level wellness was beyond even national boundaries; in the era of rapid change, no longer could health be viewed as exclusively a local phenomenon: “*The effects of these (environmental/social) changes ripple outward to all parts of the physical environment, affecting the entire ecology on which man is dependent, and also penetrating into the deepest recesses of his inner world*” [13]. The search for high-level wellness in life (Greek, bios) cannot be separated from our individual and collective mode of living (Greek, biosis) or lifestyle; to understand such connections, Dunn advocated for educational efforts to “*develop interest in biology on a vast scale, so that it would become of major interest to all. This would mean acquiring a deep interest in life*—*in the life process itself*” [14]. Related to this, Dunn emphasized a need to understand how human attitudes to other forms of life (and the natural environment in general) are formed. 

The prerequisite to individual and societal high-level wellness, Dunn contended, is the maintenance of a sense of purpose and opportunities for creative expression. On the other hand, he argued that the barriers to high-level wellness include authoritarianism, clinging to dogma, and lack of critical analysis skills. He encouraged health and medical bodies to self-reflect. Barriers to high-level wellness, Dunn argued, are manifest in uncritical allegiance to “teams” in political, economic, occupational, academic, and other professional and social spheres; in particular, the inability to adjust beliefs and communication based on advancing knowledge is a major impediment. 

Dunn maintained that global wellness in the modern era is predicated upon providing opportunities (especially early in life) to see common ground, teaching children critical appraisal skills, and learning the value of listening to opposing views while ‘searching for points of mutual agreement’. Dunn proposed a ‘universal philosophy of living’ which focused not on what individuals were ‘against’, but rather what they would be ‘for’: “*a philosophy which will permeate the minds and hearts…a philosophy which men and women of good will, regardless of race, creed and nationality, can be for. A unifying type of philosophy which can be embraced and lived by all, within their own cultural background*” [15]. 

He also called for greater research investments to be directed toward an understanding of the social, biochemical, physiological, and psychological pathways to the goal of high-level wellness; Dunn maintained that high-level wellness was itself a way of life—a lifestyle which involved a sense of purpose and meaning—one which maximized the odds of achieving the fullest potential. In its simplest form, high-level wellness equates to vitality; humans can experience the upper ranges of wellness when there is a feeling of ‘zest in life’, abundant energy, a tingle of vitality, and a feeling of ‘being alive clear to the tips of your fingers’. However, Dunn cautioned that in 20th century modernity, zest was being confused with ‘*something that gives us a very momentary “lift”*’ [14]. In the 21st century, the iron pyrite of zest and aliveness is all-too-often sold to the public in the form of “energy” drinks [16].

Vitality has since become a measurable psychological construct and the subject of intense research scrutiny. Several vitality scales have been validated (as well as vitality subscales within larger assessments such as the Profile of Mood States and the SF-36), and researchers have linked vitality to various health-related outcomes; for example, vitality is emerging as a surrogate marker of reduced risk of NCDs, psychological wellbeing, and better life-course health [17,18,19,20,21]. In line with Dunn’s commentaries, vitality is captured on scales as ‘approaching life with excitement and energy, feeling vigorous and enthused; living life as an adventure; feeling alive and activated; zest for life’. It is unclear if vitality is a cause or consequence of a healthy diet, exercise, social support, and other lifestyle habits such as spending time outdoors in natural environments—it is likely a mixture of contribution and cause [22,23,24,25]. The concept of high-level wellness may be identified in so-called blue zones where longevity, chronic disease resilience, and quality of life are found in tandem. 

## 4. Preventive Medicine, Public and Planetary Health

The term planetary health emerged from the annals of preventive medicine, health promotion and the environmental health movement; in 1972, physician ecologist Frederick Sargent II, MD advocated for a greater understanding of the interrelations between the ‘planetary life-support systems’ and health (not simply the absence of disease) [26]. In 1974, Soviet bio-philosopher Gennady Tsaregorodtsev called for novel and integrative approaches to ‘planetary public health’ [27]. He also advocated for a greater understanding of the biopsychosocial needs of humans in the context of ecosystems at micro- and macro-scales. Both writers underscored the urgent need for information-gathering and actionable steps in relation to the human health sequelae of environmental degradation—the focus on preventing unanticipated consequences (those corrosive to wellness) of human-induced changes to the natural environment.

On the environmental side of health, work of multidisciplinary scientists (especially ecology, toxicology, geography, and other environmental sciences) was folded into definitions of health by environmentalists and various advocacy groups. For example, in 1980, the environmental group Friends of the Earth expanded the World Health Organization definition of health to include ecological and planetary health inputs: “*health is a state of complete physical, mental, social and ecological well-being and not merely the absence of disease—that personal health involves planetary health*” [28]. 

At the same time, these sentiments were echoed within the growing holistic health movement of the 1980s which argued for: “*greater attention to prevention…(and) a different philosophical framework through which individual, community, environmental and planetary health can be better understood in a broad and integrated fashion*” [8]. Nursing, a profession which has been unified by deeper understandings of the words ‘health’ and ‘care’, was progressive in underscoring planetary health: “*the health of each of us is intricately and inextricably connected to the health of our planet*” [29]. By the early 1990s, leaders in nursing advocated for a need to “*understand health as a reintegration of our human relationships with nature…(and maintain) openness to nature’s healing power” (and a) “broader ecologically-informed perspective on health*” [30]. 

By the mid-1990s, the ‘wellness movement’ had, according to experts in health education, “*added a sixth dimension of health (that is, in addition to physical, social, emotional, intellectual, and spiritual), environmental or planetary, health. This dimension involves both micro (immediate, personal) and macro (global/planetary) environments*” [31]. Health education textbooks maintained that we must “*now view health as the presence of vitality—the ability to function with vigor and live actively, energetically, and fully. Vitality comes from wellness, a state of optimal physical, emotional, intellectual, spiritual, interpersonal, social, environmental, and even planetary wellbeing*” [32]. Viewed this way, the word health cannot be disassociated from the words equity, access, and opportunity.

It is also important to point out that the ‘planetary health’ movement which began in the 1980s was an extension of indigenous knowledge and ideation: scholars have underscored that indigenous cultures have long-since understood that “*human health and planetary health are the same thing*” (or “*to harm the Earth is to harm the self*”) [33]. For example, Lori Alvord, MD, the first conventionally-trained female Navajo surgeon in the United States, stated: “*I cannot think of a single thing that would be more important to us (North American indigenous peoples) than to have a pure environment for our health…human health is dependent upon planetary health and everything must exist in a delicate web of balanced relationships*” [34]. 

An understanding of the links between human and planetary health among indigenous peoples is a product of emotional bonds with the natural environment and effective, trans-generational knowledge transfer [35,36]. Indeed, the ecopsychology movement of the early 1990s advocated for “*a planetary view of mental health…to live in balance with nature is essential to human emotional and spiritual well-being, a view that is consistent with the healing traditions of indigenous peoples past and present*” [37]. 

In sum, the environmental health, preventive medicine, and wellness movements of the late 20th century often included a planetary health perspective. However, it must be recognized that the foundations of the contemporary planetary health concept are a product of indigenous science and medicine, and longstanding awareness that human health (that is, wellness) is dependent upon the vitality of the natural environment [38]. In the context of high-level wellness, preventive medicine is tasked not only with helping to prevent the path to specific diseases, but to prevent departure from vitality. We turn now to examine the accelerating pace at which the term planetary health has moved into the glossary of science and medicine. 

## 5. Planetary Health Moves to Mainstream

*“Even with all our medical technologies, we cannot have well humans on a sick planet. Planetary health is essential for the well-being of every living creature. Future healthcare professionals must envisage their role within this larger context, or their efforts will fail in their basic objective. Although until recently healthcare providers could ignore this larger context, such neglect can no longer be accepted”.*

Thomas Berry, 1992 [39]

Although the term planetary health was used frequently by various experts, researchers, clinicians, academics, and advocates, only recently has the concept entered the lexicon of mainstream science and medicine. In 2015, the Rockefeller–Lancet Commission on Planetary Health published its landmark report; the expansive document—which covered political, economic, and social systems—formally defined planetary health as “*the health of human civilization and the state of the natural systems on which it depends*”, with its stated goal to find ‘*solutions to health risks posed by our poor stewardship of our planet*’ [9]. As a crude measure of the report’s impact, results of a PubMed search for “planetary health” demonstrate that over 70% of the citations have been published post-2014. The *Commission* report, financially supported by the Rockefeller Foundation, has already been cited over 300 times on Google Scholar; it has also spawned a dedicated *Lancet Planetary Health* journal. There is little doubt that the *Commission* report and the efforts of other groups have moved planetary health into widespread discussion. 

The contemporary planetary health concept is meant to break down silos and galvanize research efforts so that there is greater awareness of how specific pieces of research work toward solving the (interrelated) grand challenges of our time; planetary health is, of course, the terrain of environmental impact assessments and strategic environmental assessments, climate indicators, and toxin-based units of analysis; however, in 2018, one of the leading voices in the current planetary health movement—Lancet Editor-in-Chief, Dr. Richard Horton—underscored that it is so much more:

“*Planetary health, at least in its original conception, was not meant to be a recalibrated version of environmental health, as important as environmental health is to planetary health studies. Planetary health was intended as an inquiry into our total world. The unity of life and the forces that shape those lives. Our political systems and the headwinds those systems face. The failure of technocratic liberalism, along with the populism, xenophobia, racism, and nationalism left in its wake. The intensification of market capitalism and the state’s desire to sweep away all obstacles to those markets. Power. The intimate and intricate effects of wealth on the institutions of society. The failure of social mobility to compensate for steep inequality. The decay of a tolerant, pluralistic, well informed public discourse. The importance of taking an intersectional perspective. Rule of law. Elites. The origins of war and the pursuit of peace. Problems of economics—and economists*” [40].

We agree with this sentiment. Indeed, the future of planetary health in the context of preventive medicine and environmental health requires a greater understanding of a ‘planetary health psyche’; by this we mean deeper insight into the ways in which emotional bonds are developed between person and place, and the collective cognitions and behaviors which have resulted in environmental degradation and ‘Anthropocene Syndrome’ in the first place [41]. This goes far beyond the now extensive research showing the health benefits—physical, emotional, cognitive, social, and spiritual health—of contact with natural environments [42,43].

The preventive form of planetary health is now an imperative; as stated by Harvard psychiatrist John E. Mack (1929–2004), we must develop a relational psychology of the Earth which allows us to “*tell unpleasant or unwelcome truths about ourselves…to explore our relationship with the Earth and understand how and why we have created institutions that are so destructive to it…we in the West have rejected the language and experience of the sacred, the divine, and the animation of nature. Our psychology is predominantly a psychology of mechanisms, parts, and linear relationships. We have grown suspicious of experiences, no matter how powerful*” [44].

The development of emotional connections with the natural world—and health-related associations with such emotional bonds—is now a measurable construct in the form of nature relatedness (see also, related validated instruments such as nature connectedness or nature connectivity scales) [45]. Nature relatedness scales are a means for researchers to evaluate individual levels of awareness of, and fascination with, the natural world; nature relatedness scores encapsulate the degree to which individuals have an interest in making contact with nature. While this body of research is far from robust, the available evidence indicates that nature relatedness is positively associated with general health, mental wellbeing, empathy, pro-environmental attitudes/behaviors, and humanitarianism (and negatively with materialism) [46,47,48,49,50,51].

The challenge for global researchers is to develop a more sophisticated understanding of how nature relatedness fits into the planetary health imperative; how is nature relatedness fostered and how is it influenced by cultural experience and socioeconomic variables [52,53]? What are the biological underpinnings of nature relatedness in relation to non-communicable disease [54]? How does it influence environmental behaviors and the political-economic viewpoints outlined by Horton [55]? Are high levels of nature relatedness a ‘burden’ in some cases? For example, in cases where environmental degradation and biodiversity losses are immediately apparent [56], it might be expected that rapidly changing environmental conditions would provoke distress (Box 2).

Box 2 **Environmental Degradation, Ecological Grief.**

Humanity is facing colossal, interconnected global challenges. It is now abundantly clear that human-caused climate change represents a threat to all of humanity. Extreme temperature and weather events, degraded air quality, and the spread of diseases via food, water, and alterations to the life of vectors (such as ticks and mosquitoes) are now a reality [57]. Climate change does not stand alone as a looming public health threat. It is coupled with environmental degradation (through industry and invasive species), biodiversity losses, grotesque health disparities, the global spread of ultra-processed foods, and what has been described as a ‘pandemic’ of non-communicable diseases [41,56,58,59]. The burden of these global threats is shouldered by the socioeconomically disadvantaged.

Only recently have researchers begun to tabulate the ways in which environmental degradation takes its toll on mental health. In areas where environmental degradation has already been significant, researchers see a worsening of mental health—described by some as ‘ecological grief’ [60]. There is an urgent need to study the ways in which climate change and environmental degradation not only contribute to NCDs, but also how they contribute to mental stress and diminish vitality [61,62,63].

## 6. Planetary Health vs. Authoritarianism

More than ever before, medicine, science, and health (at all scales) are political discussions [64,65,66,67]. A rapid change in communication technology and social media has accelerated the ability of misinformation to spread globally. We have now entered a strange era dubbed ‘post-truth’, [68], a time when it is no long tenable to be on the sidelines as a health ‘care’ spectator. However, in comparison to other professions and even the general population, US physicians show low levels of civic participation [69,70]. 

Recent elections in North America and Europe have underscored the ways in which public health is threatened by political authoritarianism [71,72]; however, authoritarianism and social dominance orientation are not constrained to the political arena and politicians. Rather, they can be found in many contemporary social structures, including those associated with westernized medicine [73] and science [74]. 

In his writings on wellness, Dunn underscored that authoritarianism is a significant barrier to global wellbeing; in order to remedy this, he encouraged greater inclusion of political science in health research and education. He also advocated for a greater understanding of leadership styles as influence on the health of groups, and broader awareness of the ways in which scientific findings are selectively misused. In particular he was concerned about the abuse of science by socially-dominant political elites and those with biased interests in the outcomes.

During Dunn’s time, the research on authoritarianism (as a psychological construct) was still in its infancy. Today, this area of research is far more robust, and it is much easier to determine the ways in which it interferes with health. Authoritarianism is described as expecting or requiring people to obey; favoring a concentration of power; limitation of personal freedoms. Scores on authoritarianism scales are associated with stigmatization of out-groups, a rigid adherence to mainstream convention, and broad aspects of prejudice [75,76,77]. Authoritarianism predicts intolerance to diversity and differing cultures, aggression toward out-group members, and hyper-vigilance to threats against non-conformism. It is also associated with a cognitive style devoid of fine-grained discourse and nuance; out-groups are labeled in simplistic, all-or-none fashion [78]. 

Social dominance orientation (SDO) is a related psychological construct that is characterized by attraction to hierarchy and areas of prestige found within social systems. SDO scales capture beliefs regarding the acceptability or entitlement of high-status groups to dominate other groups, and attitudes toward maintaining social and economic inequality. Higher scores on SDO scales are associated with lower empathy, and less concern for matters of social justice and inequalities [79]; conversely, these individuals are hyper-vigilant to threats—real and perceived—that might compromise privileged status and its benefits [80]. Researchers have shown that higher SDO predicts prejudice and diminishes awareness that power gained from the dominant social position is being used for personal gains [81,82]. The overlaps between SDO and authoritarianism have been consistently noted, such that researchers refer to the combination of SDO and authoritarianism as the “lethal union”.

The relevancy of authoritarianism and SDO to planetary health is now obvious. Authoritarianism and/or SDO predict denial of the seriousness of climate change, lower levels of environmental concern, and a hierarchical anthropocentric view of nature [83,84,85,86,87] Many public health professionals are keenly aware of the threats posed by political authoritarianism. Indeed, recent elections in North America and Europe have been a catalyst in (re)emphasizing the importance of political science in personal, public, and planetary health [88]. 

Empathic, caring, civil-minded professionals that fill the ranks of global healthcare are obligatory humanists; because so many health threats—those linked to ecosystems and the biosphere, and infectious/NCDs alike—are oblivious to national boundaries, humanist healthcare professionals are, in turn, obligatory anti-nationalists. Thus, public, preventive, and environmental health is built upon vigilance for political authoritarianism. It is understood that the misguided actions of any one nation, or even one individual, can conspire against all of humanity.

However, this does not mean that SDO or institutional authoritarianism is a problem to which science and medicine is immune. On the contrary, research shows that authoritarianism and/or SDO may be uncomfortably high among students at entrance to medical schools, increased through medical education, and reinforced at the institutional levels of medicine [89,90,91,92,93,94]; medicine in general, and the technical medical disciplines such as surgery in particular, maintain high levels of perceived status [91]. That is a problem not only for clinical care, but also for building (and maintaining) the public trust in science and medicine at-large. 

Research is beginning to tease out the motivations of students who enter medical school as they relate to money and status, and connect these to characteristics such as low agreeableness and intolerance of opposing views [95]. Since experimental studies show that manipulating social status and power (in an upward direction) increases social dominance, and that SDO can be provoked by status reminders and cues such as money [81,96,97], medicine may need to look inward and examine its commitment to the principles of planetary health. Indeed, contemporary research supports Dunn’s contention that individual (and in-group) authoritarianism is a barrier to the collective action required to support the core tenets of planetary health—that is, it blocks social rights-based movements (civil, gender, environmental, and otherwise.) [98]. 

As discussed in detail elsewhere [73], entering medical school with a high desire for social status, or with higher baseline levels of authoritarianism and social dominance orientation than societal norms—and to have such characteristics amplified through medical training and institutional structures—is at the heart of Horton’s plea [40] for a planetary health agenda designed for meaningful change. How can science and medicine challenge an unhealthy status quo if it is unwilling or unable to confront its own contextual power hierarchies [99,100]? These are concerns which permeate healthcare-at-large. Higher SDO (even among healthcare professionals who are not medical doctors) is associated with an unwillingness to engage in inter-professional education [101]. This is likely to reflect more generalized shifts in societal goals and value systems away from meaningful life philosophy towards an emphasis on financial wealth as the dominant measure of success [102].

## 7. Conclusions

The contemporary concept of planetary health—which has its roots in the late-20th century preventive medicine and environmental health movements—emphasizes that health equates to vitality at scales of person, place, and planet. It asserts that preventive medicine is a broad term, one which extends to the planet’s natural systems—the ecosystems and biodiversity upon which our own vitality depends. Planetary health is an adisciplinary unifying concept which allows researchers working in seemingly disparate branches of science and medicine to understand the relevancy of the toil provided by each group. 

Specifically, we must advance the cause of planetary health by demonstrating a willingness to engage with and promote other disciplines. To this end, there are now encouraging examples of collaborative initiatives between health providers, regenerative agriculturalists, and local communities—notably developing regions of the world—with demonstrated community-wide benefits for health, wealth, employment, and environmental sustainability [103]. These integrative models provide a path forward for ensuring the health of people and planet.

In the context of planetary health, the urgent task for preventive medicine and environmental health is to provide deeper insight into the ways in which we develop relationships with nature, and how we feel, think, and respond to the natural world. This includes the biological, social, political, and economic underpinnings of nature relatedness (and related psychological constructs) and its impact on vitality at all scales. It includes a more fine-grained understanding of what prevents the planetary health goals set forth by the WHO and the Lancet Commission on Planetary Health report [9]. From our perspective, this means further study of authoritarianism and social dominance orientation (at individual, institutional and other scales) vis à vis the structures—including those found in politics, science, medicine, and elsewhere—which either support the status quo, or provide meaningful solutions to planetary health objectives. This applies equally to the injustices and inefficiencies of global systems, such as food and international trade systems, which also serve to undermine health and equality through biased authoritarian and neoliberal ideologies [104,105].

The idea that threats to the health of the person, the place (community), and the planet are distinct from each other is a mirage; this false notion has been challenged by environmental health and preventive medicine for decades. We have moved past the point at which such discourse is merely intellectual fodder. We argue that in 2019, one simply cannot claim to be a ‘health’ care professional without advocating forcefully for the planet. There are no healthy people on an uninhabitable planet, and we are fast heading there. If its true goals are realized, environmental health and preventive medicine at the planetary scale will, as Jonas Salk implored in 1984, place emphasis on the idea that we should want “*those who follow us to look back on us as having been wise ancestors, good ancestors*” [106].
